# Transcriptome Analysis of Plant Hormone-Related Tomato (*Solanum lycopersicum*) Genes in a Sunlight-Type Plant Factory

**DOI:** 10.1371/journal.pone.0143412

**Published:** 2015-12-01

**Authors:** Yusuke Tanigaki, Takanobu Higashi, Kotaro Takayama, Atsushi J. Nagano, Mie N. Honjo, Hirokazu Fukuda

**Affiliations:** 1 Department of Mechanical Engineering, Graduate School of Engineering, Osaka Prefecture University, Sakai, Osaka, Japan; 2 Department of Applied Life Sciences, Graduate School of Life and Environmental Sciences, Osaka Prefecture University, Sakai, Osaka, Japan; 3 Faculty of Agriculture, National University Corporation Ehime University, Matsuyama, Japan; 4 Faculty of Agriculture, Ryukoku University, Otsu-shi, Shiga, Japan; 5 JST PRESTO, Honcho, Kawaguchi, Saitama, Japan; 6 Center for Ecological Research, Kyoto University, Hirano, Otsu, Shiga, Japan; Institute of Genetics and Developmental Biology, Chinese Academy of Sciences, CHINA

## Abstract

In plant factories, measurements of plant conditions are necessary at an early stage of growth to predict harvest times of high value-added crops. Moreover, harvest qualities depend largely on environmental stresses that elicit plant hormone responses. However, the complexities of plant hormone networks have not been characterized under nonstress conditions. In the present study, we determined temporal expression profiles of all genes and then focused on plant hormone pathways using RNA-Seq analyses of gene expression in tomato leaves every 2 h for 48 h. In these experiments, temporally expressed genes were found in the hormone synthesis pathways for salicylic acid, abscisic acid, ethylene, and jasmonic acid. The timing of CAB expression 1 (TOC1) and abscisic acid insensitive 1 (ABA1) and open stomata 1 (OST1) control gating stomata. In this study, compare with tomato and *Arabidopsis thaliana*, expression patterns of TOC1 have similarity. In contrast, expression patterns of tomato ABI1 and OST1 had expression peak at different time. These findings suggest that the regulation of gating stomata does not depend predominantly on TOC1 and significantly reflects the extracellular environment. The present data provide new insights into relationships between temporally expressed plant hormone-related genes and clock genes under normal sunlight conditions.

## Introduction

Cultivation environments comprise various determinants of yield and quality in agriculture, and circadian rhythms can be considered a critical component. Accordingly, circadian rhythms reportedly form the basis of the biological phenomenon that affects growth and quality of plants. However, complexities of the related biological phenomena have hampered the identification of specific determining factors, warranting analyses of chronological gene expression changes in a plant factory, and specific examinations of plant hormone-related genes that affect growth and quality.

Constant environmental conditions are not achievable for outdoor crops, and agricultural chemicals are often necessary for controlling diseases and herbivory. Moreover, despite the importance of regional agriculture, many land areas are unsuitable for agriculture due to low moisture and extreme temperatures. Thus, plant factories are under consideration as effective tools for accommodating food requirements of increasing human populations. Both closed- and sunlight-type plant factories allow control of the environment and limit the risk of disease and herbivory, leading to high yield crops. In addition, a recent plant-factory study demonstrated that selection of valuable strains during the early stages of growth can significantly improve productivity, and quality was predicted using weather, temperature, and solar radiation data [1, 2]. In contrast, such predictions are not possible under conditions of extreme weather, disease, and herbivory. In addition, because data collection is likely difficult for the general farmer, it is necessary to design analytical procedures that provide predictive power with data from simple sampling techniques.

Plant phenotypes are highly dependent on cultivation conditions, and corresponding changes in gene expression are strongly correlated with multiple plant hormone-related processes, including gating stomata and flower initiation. In particular, clock genes are reportedly involved in the majority of these processes [3]. Clock genes form circadian rhythms, often with 24-h cycles, and comprise conserved mechanism under constitutive conditions [4]. In plants, circadian rhythms are reportedly regulated by photosynthesis, gating of stomata, cell elongation, and flower initiation. Although clock genes have been associated with transcription–translation feedback loops, the related mechanism remains unknown [5].

The plant hormones salicylic acid (SA), abscisic acid (ABA), ethylene (ET), and jasmonic acid (JA) are reportedly stimulated by changes in environment. Among these, SA is produced under conditions of disease and/or injury and induces cell death following direct induction via the proteins phenylalanine ammonia-lyase (PAL), phytoalexin deficient 4, enhanced disease susceptibility 1, and nonrace-specific disease resistance 1 [6–8]. Synthesis of SA is promoted by ET [9], and methylated SA (MeSA) transduces signals to neighboring plants [10]. ABA production involves 9-*cis*-epoxycarotenoid dioxygenase, abscisic acid deficient 2 (ABA2), and abscisic aldehyde oxidase 3 (AAO3), which are induced by and confer resistance to drought and salt stress [11]. In addition, ABA is induced by disease and injury and suppresses apoptotic SA signaling [12]. On the other hand, the ABA is involved in gating stomata. It is reported that ABI1 and OST1 are involved in the gating stomata as major genes [13]. In the non-presence of ABA, ABI1 interact with OST1 and inhibit OST1 activity. In the presence of ABA, ABI1 activity is inhibited by the binding of the ABA and ABA receptors. The plant hormone JA is associated with wound stress via the lipoxygenase (LOX) pathway and induces the production of antibacterial proteins. Subsequently, JA is methylated and, similar to MeSA, comprises a signal transduction molecule that affects the whole plant body and neighboring plants [14]. ET not only has various biological effects during responses to environmental stress, disease, and/or injury but also plays roles in germination and growth [15, 16]. Hence, these hormone responses require accurate control as survival responses. In a recent study of plant hormone mechanisms, SA was shown to antagonize ABA, and the downstream responses reflected cellular SA/ABA ratios [17, 18]. In contrast, JA production is promoted by ET signaling, which suppresses SA signals [19]. These hormone pathways lead to the expression of genes that suppress other pathways, suggesting that each hormone is intricately regulated by interactions and cross talk between environmental and growth response variables. Among these, it is reported that the clock gene pattern recognition receptors (PRRs) regulated responses to cold stress and the clock gene circadian clock-associated 1 (CCA1) which has same functions as late elongated hypocotyl (LHY) was associated with reactive oxygen species [20, 21]. However, intricacies of the relationships between plant hormones and clock genes remain unknown. Thus, in the present study, we performed transcriptome analysis and examined patterns of plant hormone gene expression under normal sunlight conditions to elucidate relationships between circadian rhythms and plant hormones, and we attempted the prediction of phenome from the analysis result. In the prediction of phenome using gene expression analysis, such as the transcriptome analysis, genetic information is very important. However, most of the crops cultivated in the general agriculture and plant factories are non-model plants. In addition, in agriculture, a commercial analysis method that is low cost and provides quick results is important. Therefore, we used the genetic information of *A*.*thaliana* of the model plant, in addition to its homology with tomato and the temporal expression patterns of the gene and its related genes.

## Materials and Methods

### Plant material

**Fig 1:**
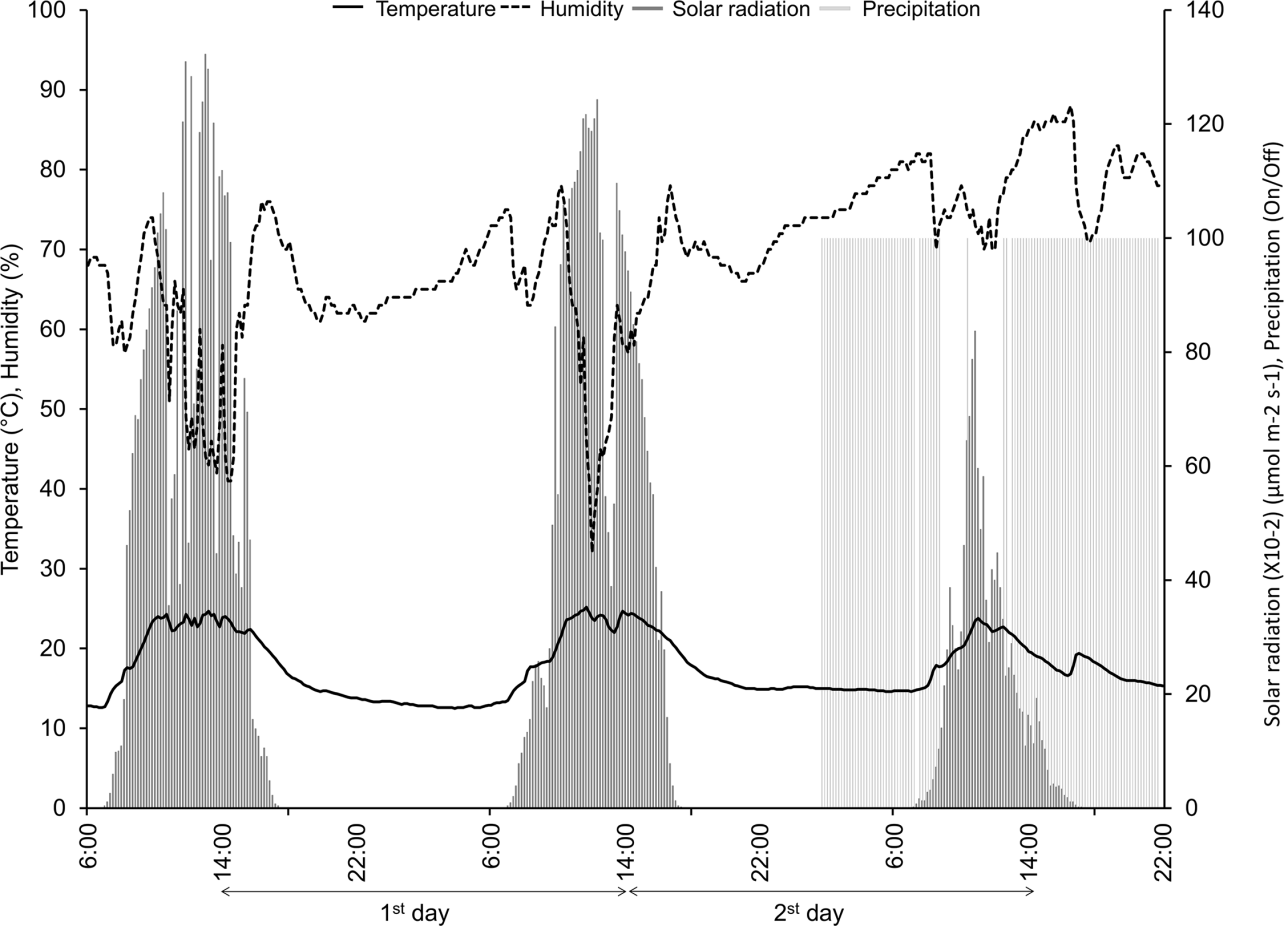
Environmental conditions in a sunlight-type plant factory. Environmental conditions in Matsuyama, Japan, on January 6^th^–8^th^, 2014. Measured values were recorded from instruments installed in the plant factory. Temperature was calculated as the average of two measurements. Temperature (black line), relative humidity (black broken line), solar radiation (gray bar), and precipitation (light gray bar) are presented in 10-minute intervals. The arrows show experimental periods, and daily measurements were recorded between 14:00 and 14:00.

The tomato cultivar Taian-kichijitsu was grown on soil in a semi-commercial sunlight-type plant factory at the Ehime University, and the temperature, relative humidity, and amounts of insolation were recorded as shown in Fig 1. Temperature was maintained at 14°C between 18:00 and 8:00. Additionally, the tomato cultivar Taian-kichijitsu was grown in a fully controlled plant factory using a deep flow technique hydroponic system. Cultivation medium was prepared and used as described by Higashi *et al* [22], and cultures were grown at 22°C with 50% relative humidity and continuous light [250–450 μmol m^−2^ s^−1^ (PPFD)].

Tomato cultivation in LL condition (continuous light), tomato seeds were grown under 12-h light/12-h dark conditions in fully controlled plant factories. Seedlings were planted after 7 days and were grown for 14 days. Subsequently, light conditions were changed to the LL condition, plants were cultured for 1 day, and sampling was performed on the second day.

### Extraction of RNA

Superior Tomato leaves that have been cultivated in sunlight-type plant factory and LL condition were sampled every 2 h for 2 days, and sliced sample leaf segments of 1 cm^2^ were stored at 0°C in RNA-later solution (QIAGEN, Venlo, Limburg, The Netherlands). Total RNA was extracted from each sample using an Agilent Plant RNA Isolation Mini Kit (Agilent Technologies, Santa Clara, CA, USA) according to the manufacturer’s instructions. RNA quantity was determined using a Bioanalyzer (Agilent Technologies).

### RNA-Seq library preparation and sequence analyses

The RNA-Seq library was prepared as previously described [23, 24]. Briefly, the extracted total RNA was mixed with ERCC RNA Spike-In control mixes (Life Technologies, Carlsbad, CA, USA) and a selective depression RNA oligo pool (see reference 24). The mixture was annealed using a thermal cycler and DNA/RNA hybrid products were digested using a Hybridase Thermostable RNase H (Epicentre, Illumina, San Diego, CA, USA). Purified RNA was obtained from the mixture after treatment with DNase I (Takara, Otsu, Japan) and digested selective depression RNA oligo pool. Purified RNA was fragmented by heat treatment, and reverse transcription was performed using M-MuLV Reverse Transcriptase (Enzymatics) and random hexamers. Second strands were synthesized using DNA polymerase I, and dsDNA ends were repaired using an End-Repair Mix LC (Enzymatics) and dA-tails were added using Klenow 3′→5′ exo- (Enzymatics). A-tailed DNA was added to the Y-shape adapters 5′-A*A*TGATACGGCGACCACCGAGATCTACACTCTTTCCCTACACGACGCTCTTCCGAT *C*T-3′ and 5′-/5P/-G*A*TCGGAAGAGCACACGTC TGAACTCCAGTC*A*C-3′ (asterisk indicates phosphorothioate bonds and 5P indicates phosphorylation) using T4 DNA Ligase (Enzymatics), and small (<200 bp) and large (>600 bp) fragments were removed using gel extraction.

The RNA-Seq library sequence analyses were entrusted to BGI (Shenzhen, China). Subsequently, we used a HiSeq 2000 sequencer (single end, 50 bp; Illumina) to obtain read files.

### Transcriptome analysis

The quality control of clean reads used FastQC software (http://www.bioinformatics.babraham.ac.uk/projects/fastqc/). Clean reads were analyzed for mapping against 1706 genome sequences from the reference tomato Heinz, and mapping data were quantified using the RNA-Seq by Expectation Maximization (RSEM) software (http://deweylab.biostat.wisc.edu/rsem/) with Bowtie2 software (http://bowtie-bio.sourceforge.net/bowtie2/index.shtml.) [25]. RSEM software calculates expression values based on the lengths of target contigs and numbers of mismatches, and isoform- or gene-level estimates were obtained using EM algorithms and read counts for each contig. Accordingly, accurate high estimates of expression data were provided even for data that was mapped at multiple points. The mapping parameter in Bowtie2 was set to default. The expression data were normalized to the RPKM value. Expression data were registered with the DDBJ database (http://trace.ddbj.nig.ac.jp/DRASearch, Accession numbers; DRA003530, DRA003529 and DRA003528). Histogram analyses with RPKM values of <0.1 were set to 0.1, and RPKM values were log (2) transformed. Statistical analyses were performed using the GeneCycle and samr packages of R software. *p*-values were calculated using Fisher’s exact *g* test, and sorting data for heatmaps were as follows: 24-h cycle; maximum value, 7 (time, 6:00); minimum value, 1 (time; 18:00), 6 per 12-h changes. Transcriptome data were sorted using the Genefilter package of R software. A heatmap was generated using the stats package and R software. Mapping analyses were performed using MapMan software (Ver.3.5.1) with a BIN code that was assigned by Mercator (http://mapman.gabipd.org) based on the TAIR10 date (http://www.arabidopsis.org) of *A*. *thaliana*. The BLAST cutoff value of Mercator was set at 50. The categorization of temporally expressed genes with *p*-values of <0.05 and FDR values of <0.05 was performed using MapMan software.

### Search for homologous genes

Searches for hormone pathway genes in *A*. *thaliana* were performed using the NCBI database (http://www.ncbi.nlm.nih.gov/), the TAIR database (http://www.arabidopsis.org/), and the Phytozome database (http://www.phytozome.net/, Ver. 9.1; S1 Table). Subsequently, searches for homologous tomato hormone-related genes were performed from transcriptome data using the BLAST system. Motifs and domains of homologous tomato genes were identified using the Panther (http://www.pantherdb.org/) and Pfam (http://pfam.xfam.org/) databases of predicted amino acid sequences.

## Results

### 1516 genes were expressed temporally

**Fig 2:**
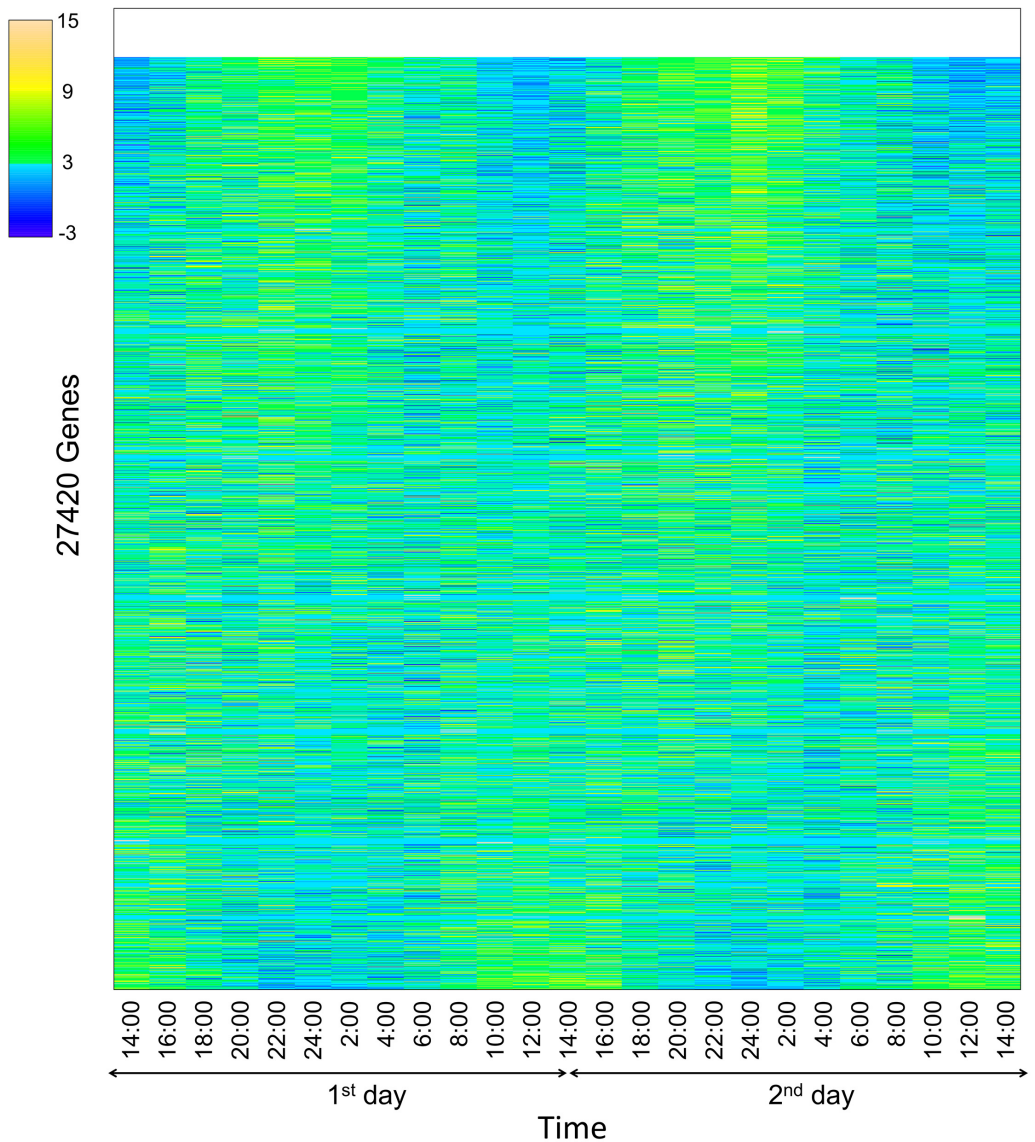
Heatmap of all expressed genes. Gene expression was calculated using log-transformed RPKM values. Gene expression data were rearranged according to artificial data. The color palette was that of the topo.colors package of R software and shows data in 12 steps. Correlation coefficients are listed in order of gene (high, A). Genes with log values of ≤0.1 were used as blanks (1090 genes).

**Fig 3:**
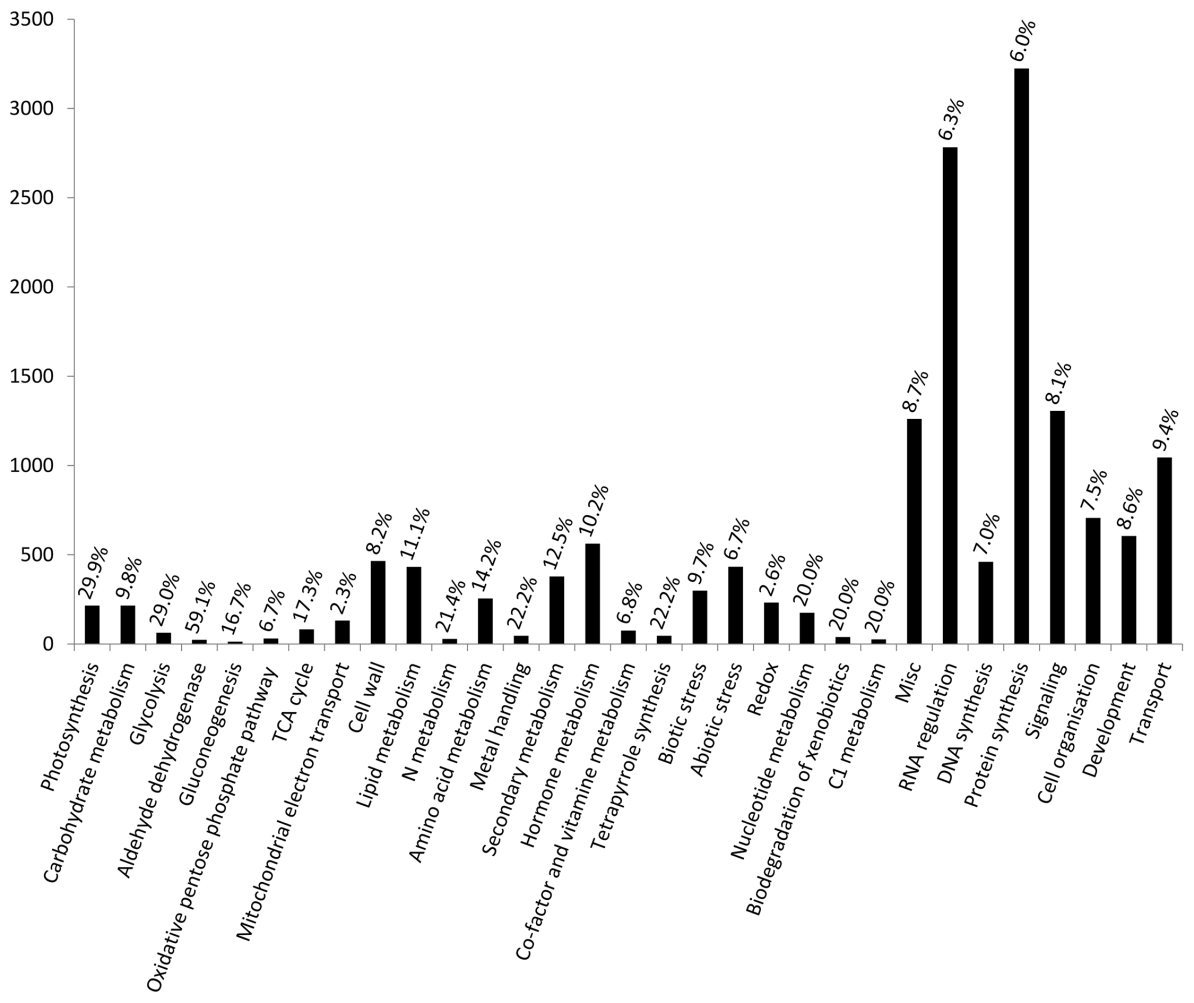
Categorization of expressed genes. Classifications were performed using MapMan software (Ver. 3.5.1) based on *Arabidopsis thaliana*. Duplicate mapping genes were not included. *p-values* and false discovery rate (FDR) values were calculated using the GeneCycle package of R software. Temporally expressed genes with *p*-values of <0.05 (Fisher’s exact *g* test) and FDR values of <0.05. Duplicate mapping genes were not included; numbers on the graph indicate the genetic rates of temporal expression patterns in all genes of each category.

RNA-Seq analyses of gene expression in tomato leaves were conducted every 2 h for 48 h. From 2:00 on the second day, rainfall reduced solar radiation and diminished further rises in humidity (Fig 1). We sampled tomatoes leaves in January, and the tomatoes were harvested. Quality control analyses showed that all clean reads had a quality of more than Q30. In initial experiments, expression data were collected for 27,420 genes, and patterns of expression were analyzed using heatmaps, which showed temporal expression patterns of multiple genes, and very low expression levels of a few genes (blank of Fig 2). Temporally expressed genes were predominantly detected in the daytime or the evening, and many were up-regulated in the evening. Subsequently, MapMan software was used to categorize all genes based on homology with *A*. *thaliana*, and after removing overlapping genes, 15,660 genes were functionally categorized (Fig 3; Table 1). Among these, 3223 genes were mapped to protein synthesis and 2782 genes were mapped to RNA regulation. Smaller groups included genes of gluconeogenesis (12 genes), sulfur assimilation (13 genes), and micro RNA (1 gene).

**Table 1:** Statistical analysis of hormone pathway-related genes.

			First-time		Second-time	
Pathway	Name	Accession number	*p*-value	FDR	*p*-value	FDR
ABA	NCED1	NM_001247526	0.598470941	1	0.417218509	1
	ABA2	XM_004237780	0.004390867	0.164600779	0.002415489	0.01261991
	ABA3	NM_001247215	0.228358254	1	0.242356502	1
	AAO3	XM_004228420	0.040410832	0.646466136	0.00175458	0.010106046
	RD22	NM_001247592	0.250800104	1	0.732093357	1
	ABI1*	XM_004253043	5.52x10^-10^	6.82x10^-7^	6.75x10^-11^	1.40x10-6
ET	ACO	NM_001247095	0.000944386	0.045404865	0.017391863	0.126222321
	ACS1	NM_001246993	0.561903626	1	0.759112037	1
	ERF1*	NM_001247912	0.754991246	1	0.000691645	0.047272975
	EIN2	NM_001247589	0.00179103	0.083578781	0.052872935	1
	EIN3	NM_001247002	0.000329246	0.022856563	0.008732967	0.126222321
	PDF1	XM_004243183	0.163897973	1	0.705186156	1
	CTR1	NM_001247525	0.26534532	1	0.416235351	1
	ERF4	NM_001247384	0.121501125	1	0.00606956	0.041799004
SA	PAL*	XM_004246603	0.000116372	0.014277805	1.48x10^-05^	0.00817534
	PAD4	XM_004233289	0.002478203	0.103101797	0.025307983	0.730327731
	EDS1	XM_004241459	0.008421372	0.270309109	0.001088403	0.010106046
	NDR1	XM_004228677	0.31725212	1	0.574025558	1
	NPR1	XM_004249262	0.663740245	1	0.416362571	1
	EDR1	XM_004245317	0.454908238	1	0.152444619	1
JA	LOX	NM_001247944	0.440427965	1	0.999119933	1
	AOS*	XM_004251113	0.001094034	0.058522363	0.000910357	0.00817534
	AOC	NM_001247090	0.158238543	1	0.610437915	1
	OPR1*	NM_001247852	1.36x10^-06^	0.00031835	9.18x10^-08^	0.000224763
	VSP	XM_004235589	0.046045798	0.654286815	0.742223864	1
	WRKY51*	XM_004245017	0.001203042	0.058522363	0.003244087	0.041799004
	JAI1	XM_004245847	0.702639509	1	0.293824715	1

Names indicate homologs of *Arabidopsis thaliana* genes in *Solanum lycopersicum*. Homology was predicted using information from the Pytozome database (Ver. 9.1), the NCBI database, the Pfam database (Ver. 27.0), and the Panther database (Ver. 9.0). *p* and FDR values were generated using R software (Ver. 3.1.1) with the GeneCycle package, and *p*-values were calculated using Fisher’s exact *g* test. Asterisks indicate temporally expressed genes with *p*-values of <0.05 and FDR values of <0.05. Significant *SI_ERF1* and *Sl_AOS* expressions were not detected in the first experiment. *WRKY51* expression was insufficient for statistical analyses in both experiments. ABA, abscisic acid; ET, ethylene; JA, jasmonic acid; SA, salicylic acid.

Evaluations of temporal expression of all genes were revealed 1516 genes with 24-h period expression patterns (*p*-values of <0.05 and false discovery rate (FDR) values of <0.05). Subsequently, 1516 temporally expressed tomato genes were functionally categorized based on *A*. *thaliana* in a mapping file using MapMan software followed by Mercator software. These analyses identified functions of 1516 genes (Fig 3), including 193 genes that were mapped to protein synthesis and 175 genes that were mapped to RNA regulation but failed to categorize 344 genes. In addition, three to seven genes were mapped to aldehyde dehydrogenase, nitrogen metabolism, sulfur assimilation, polyamine metabolism, and xenobiotic degradation pathways. Categorized as photosynthesis, glycolysis, and tetrapyrrole metabolism genes, respectively, more than 20% were expressed temporally (Fig 3). Moreover, about 10% of genes involved in secondary metabolism, hormone metabolism, biotic stress, and abiotic stress pathways were expressed temporally, and these were included as responsive genes in further analyses.

### Temporal expression of responsive genes

**Fig 4:**
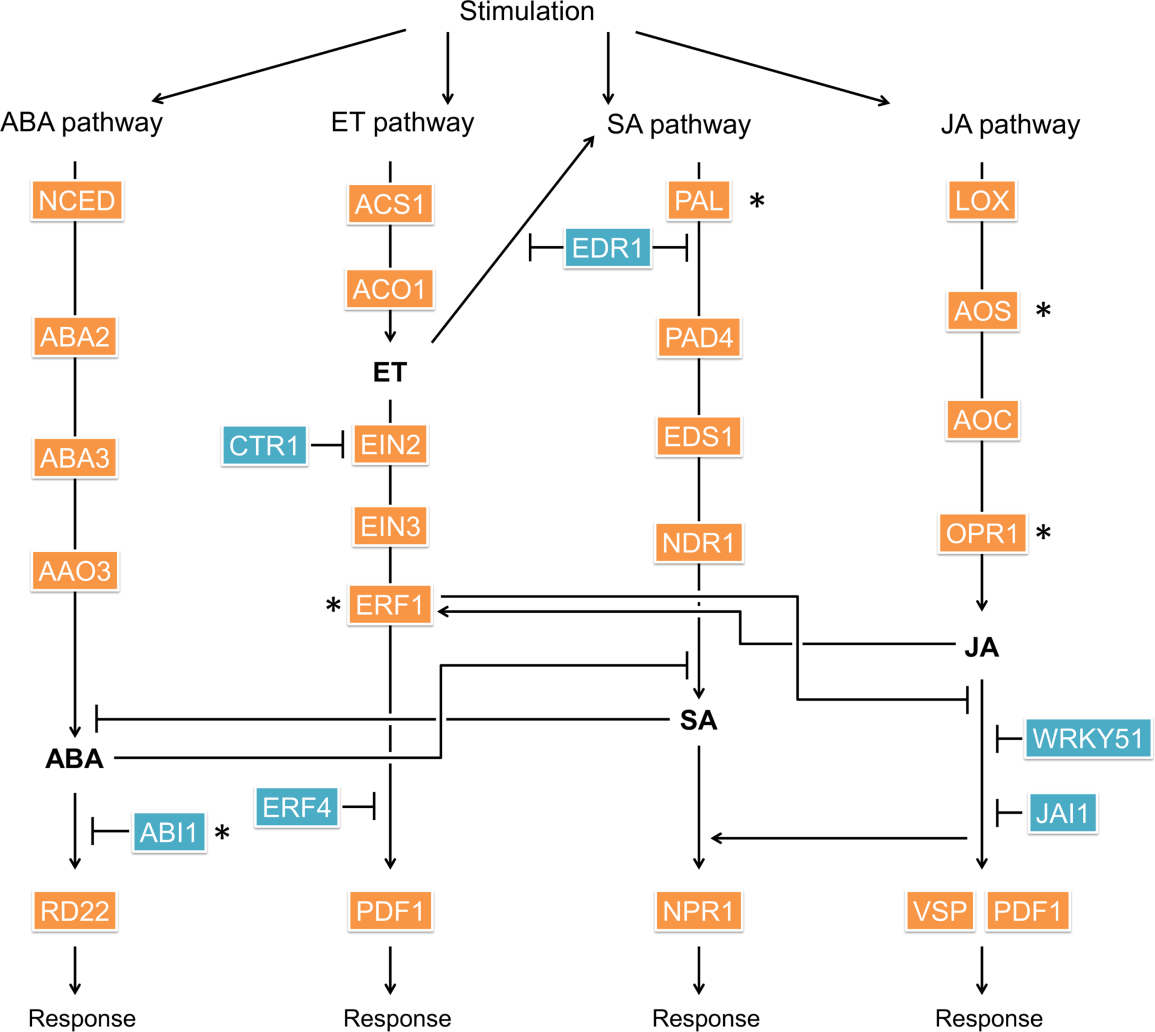
Overview of prediction plant hormone pathway responses. Abscisic acid (ABA), ethylene (ET), salicylic acid (SA), and jasmonic acid (JA) are representative plant hormones and are known to respond to various stimuli. Orange and blue boxes show representative hormone-related genes with homology to those in *Arabidopsis thaliana* (S1 Table). Orange boxes indicate biosynthetic products; blue boxes indicate genes that suppress hormone pathways. Asterisks (*) indicate temporally expressed genes with *p*-values of <0.05 and false discovery rate values of <0.05. ABA pathway: SDR, short-chain dehydrogenase/reductase; ABA3, abscisic acid deficient 3; ET pathway: ACO1, 1-aminocyclopropane-1-carboxylate oxidase; ACS1, arabidopsis cysteine synthase 1; EIN2, ethylene insensitive 2; PDF1, plant defensin 1; CTR1, constitutive triple response 1; ERF4, ethylene response factor4; SA pathway: PAL, phenylalanine ammonia-lyase; SID2, salicylic acid induction deficient 2; EDR1, enhanced disease resistance 1; NPR1, nonexpressor of pathogenesis-related genes 1; JA pathway: AOC, allene oxide cyclase; VSP, vegetative storage protein; JAL1, jasmonate resistance long hypocotyl 1. WRKY is a transcription factor.

Plants are highly sensitive to environment, disease, and injury stress. Moreover, plant hormone pathways have been shown to be responsive to these stimuli following induction of complex regulatory mechanisms. Previous reports of *A*. *thaliana* provide overviews of various hormone regulatory pathways, including for ABA, ET, SA, and JA (Fig 4). In the present study, homologs of hormone-related genes were predicted in the tomato database of NCBI using the BLAST system, and genetic identity with the abundantly characterized *A*. *thaliana* was confirmed. These amino acid sequence analyses showed the presence of all hormone pathway-related genes (Fig 4; Table 1) according to characteristic motifs and domains, and changes in expression data were compared over 2 time points. Among genes of the ABA pathway, *Sl_AAO3* (XM_004228420) and *Sl_ABI1* (XM_004253043) were expressed temporally (*p* < 0.05 and FDR < 0.05; Table 1), and the tomato ET pathway gene *Ethylene response factor 1* (*Sl_ERF1*, XM_004234188) and Allene oxide synthase (*Sl_AOS*, XM_004251113) was also temporally expressed, although its expression was not detected in initial experiments. The SA pathway gene *Sl_PAL* (XM_004239612) and the tomato JA pathway genes *allene oxide synthase* (*Sl_AOS*, XM_004251113) and 12-oxo-phytodienoic acid-10,11-reductase (*Sl_OPR1*, NM_001247852) were expressed temporally.

**Fig 5:**
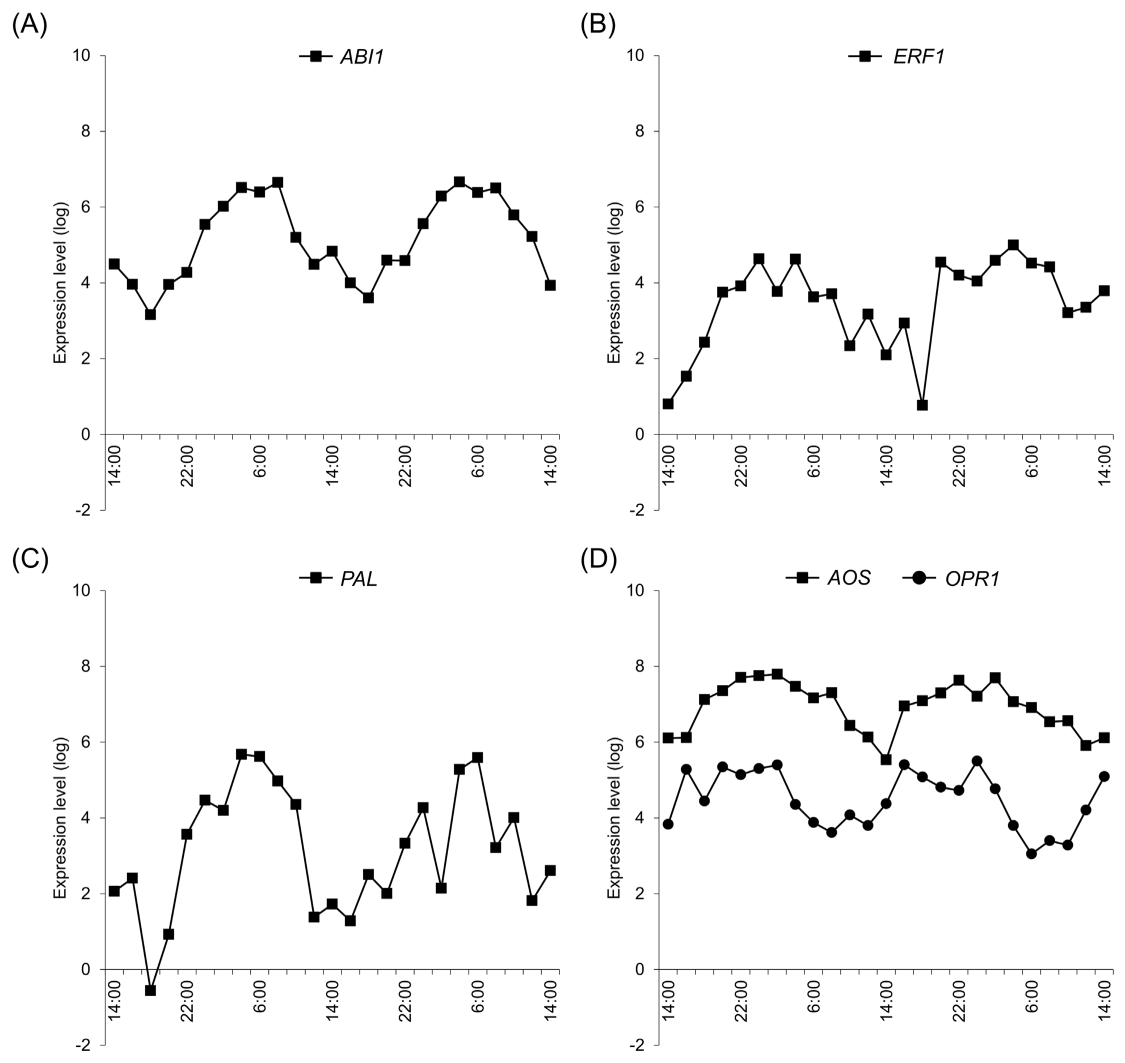
Expression patterns of temporally expressed genes. (A) Abscisic acid pathway, (B) Ethylene pathway, (C) Salicylic acid pathway, and (D) Jasmonic acid pathway.

Because at least one of these temporally expressed genes was located upstream of the hormone in all predicted hormone pathways, we performed further temporal expression experiments. In the ABA pathway, *Sl_ABI1* had a 24-h expression cycle (18:00–18:00) (Fig 5), whereas the ET pathway gene *Sl_ERF1* had a 28-h expression cycle (14:00–18:00). In the SA pathway, *Sl_PAL* had a 24-h expression cycle (18:00–18:00) and the JA pathway genes *Sl_AOS* and *Sl_OPR1* had 24- (14:00–14:00) and 22-h (6:00–8:00) expression cycles, respectively.

**Fig 6:**
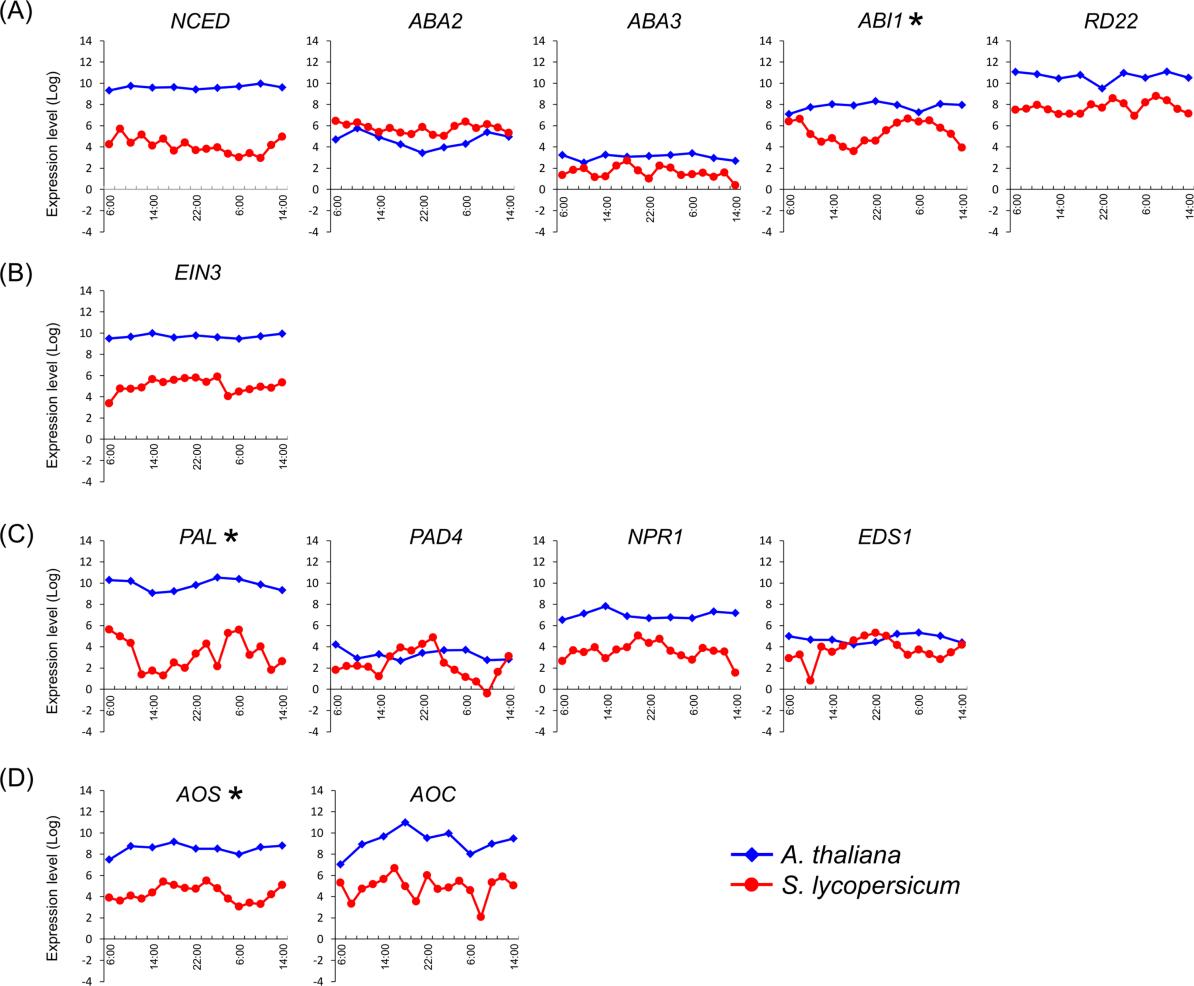
Comparison of hormone pathway-related genes in tomato and *Arabidopsis thaliana*. Temporal expression data for *A*. *thaliana* were collected from the diurnal database (http://diurnal.mocklerlab.org/) using LDHC data. *AAO3*, *ACS1*, *ACO1*, *EIN2*, *ERF1*, *PDF1*, *CTR1*, *PDF1*, *NDR1*, *EDR1*, *NPR1*, *LOX*, *OPR1*, *WRKY51*, *JAI1*, and *VSP* of *A*. *thaliana* were not temporally expressed. Gene IDs of *A*. *thaliana* are listed in S1 Table. *A*. *thaliana* genes indicated in Fig 5 that were not registered with the diurnal database were excluded from analyses. The diamond-shape (blue line) and circle-shape (red line) indicate expression patterns of *A*. *thaliana* and tomato, respectively. Asterisks indicate temporally expressed genes in tomato. (A) Abscisic acid pathway, (B) Ethylene pathway, (C) Salicylic acid pathway, and (D) Jasmonic acid pathway.

**Fig 7:**
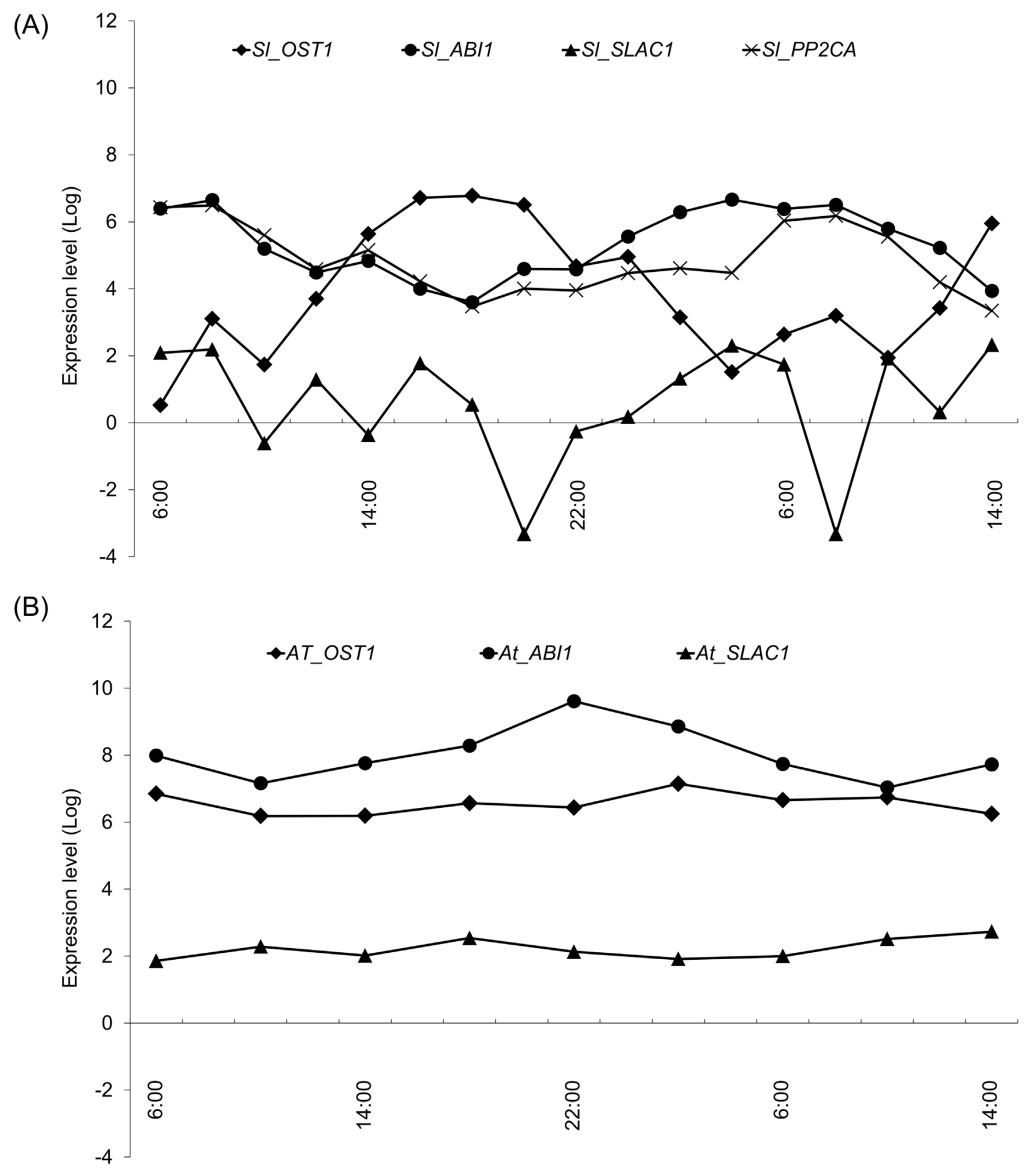
Expression levels of opening–closing stomata-related genes. Gene data for *Arabidopsis thaliana* was collected from the TAIR database (http://www.arabidopsis.org/), and relationships between ABI1 and stomata-related genes were elucidated using STRING software (Ver. 9.1). Because registered data from the diurnal database for every 4 hours from 6:00 to 2:00, we used the data of *A*. *thaliana* in the overlaped time with tomato. Expression patterns of *A*. *thaliana* genes were collected from the diurnal database using LDHC data (*At_OST1*, AT4G33950; *At_ABI1*, AT4G26080; *At_SLAC1*, AT1G12480). Homologous tomato genes were identified using the Homolog search systems in Pytozome and NCBI databases. Diamonds, triangles, circles, and x marks indicate expression patterns of *OST1*, *ABI1*, *PP2CA*, and *SLAC1*, respectively; *At_PP2CA* was not registered with the diurnal database. (A) Stomata-related genes of tomato and (B) stomata-related genes of *A*. *thaliana*.

**Fig 8:**
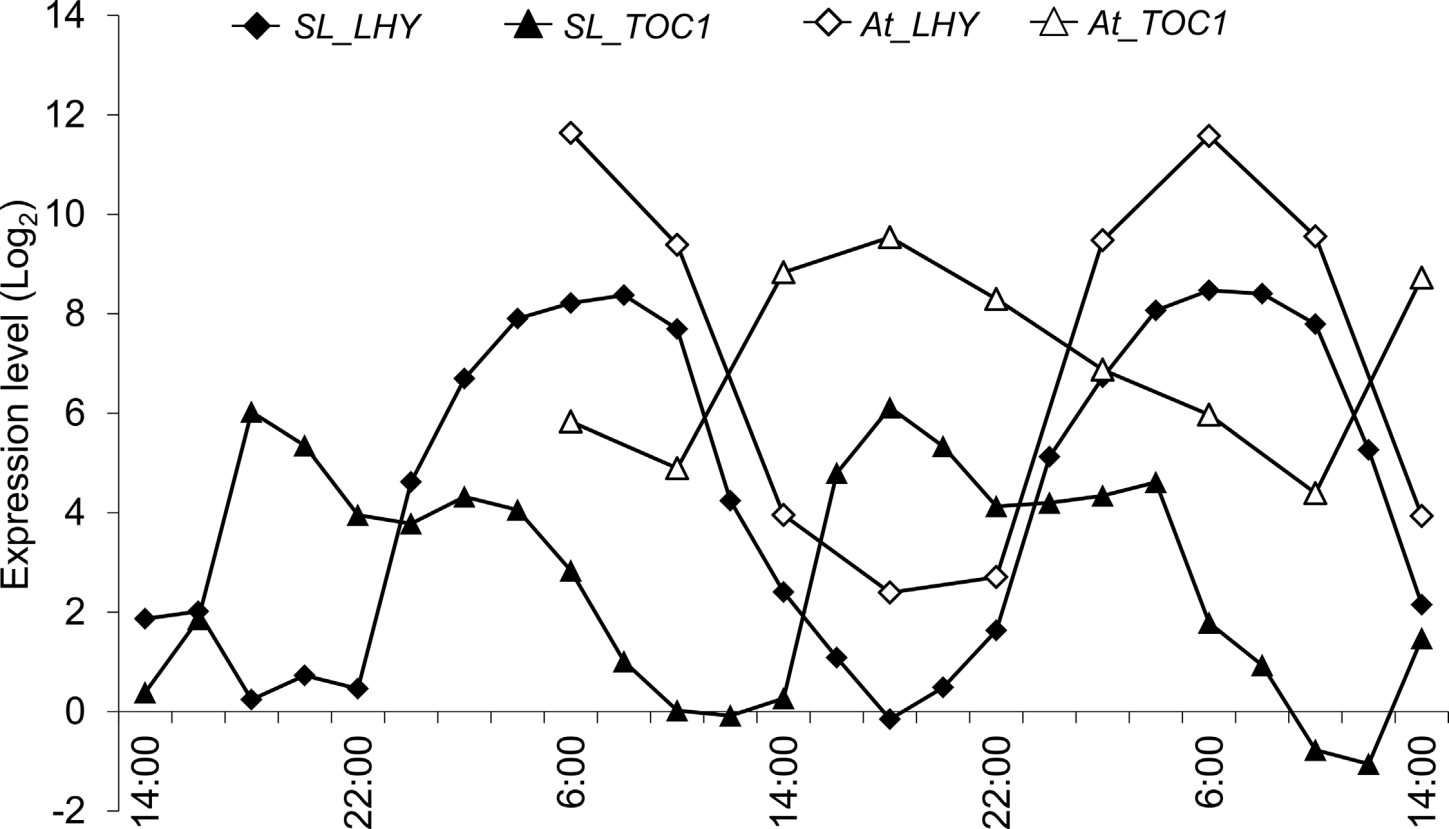
Expression patterns of *timing of CAB expression 1* (*TOC1*) and *late elongated hypocotyl* (*LHY*). *Arabidopsis thaliana* gene data were collected from the diurnal database. Registered data from the diurnal database for every 4 hours from 6:00 to 2:00, we used the data of *A*. *thaliana* in the overlaped time with tomato; diamonds, triangles, circles, and x marks indicate expression patterns of *Sl_LHY1*, *Sl_TOC1*, *At_CCA1*, and *At_TOC1*. Black and red lines indicate genes of tomato and *A*. *thaliana*, respectively.

Here, we predicted the tomato genes from a public database and examined its temporal expression along with that of its related genes. Assuming same functions of these genes, it is believed that there is a high similarity in the control mechanisms and expression patterns of the genes [26]. In further analyses, we collected temporal expression data from the diurnal database (http://diurnal.mocklerlab.org/) for *A*. *thaliana* and compared expression patterns of hormone pathway-related genes with those of tomato. These analyses showed similar expression patterns for all genes except for *abscisic acid insensitive 1* (*ABI1*) and *responsive to dessication 22* (*RD22*; Fig 6). Moreover, clear reciprocal temporal expression of *Sl_ABI1* and *At_ABI1* (AT4G26080) was observed. ABI1 is involved in the gating mechanism of stomata [13] in unstressed plants, and *open stomata 1* (*OST1*, *Sl_OST1*; XM_004232007, *At_OST1*; AT4G33950), *protein phosphatases type 2C* from group A (PP2CA, *Sl_PP2CA*; XM_004241163), and *slow anion channel-associated 1* (*SLAC1*, *SL_SLAC1*; XM_004245638, *At_SLAC1*; AT1G12480) are also associated with this mechanism. Although the expression pattern of *At_PP2CA* was not available from the diurnal database for *A*. *thaliana*, temporal expression of the Ca anion channel activator *Sl_SLAC1* did not differ between tomato and *A*. *thaliana*. *ABI1* and *PP2CA* suppress the expression of *OST1* and had similar expression patterns in both species (Fig 7), whereas the expression pattern of *OST1* was negatively associated with those of *ABI1* and *PP2CA* in tomato only. In a previous study, expression of the *timing of CAB expression 1* (*TOC1*) gene was regulated by *LHY/CCA1*, and ABA related genes were significantly induced in TOC1 mutant (toc1-2 and TOC1 overexpression), and TOC1 mutant responded sensitive to ABA [27]. It is indicated that TOC1 affects the ABA biosynthesis. Accordingly, the expression patterns of *Sl_TOC1* and *Sl_LHY1*, and *At_TOC1* and *At_LHY* were similar in the present experiments (Fig 8).

### Loss of temporal expression under conditions of continuous light

**Fig 9:**
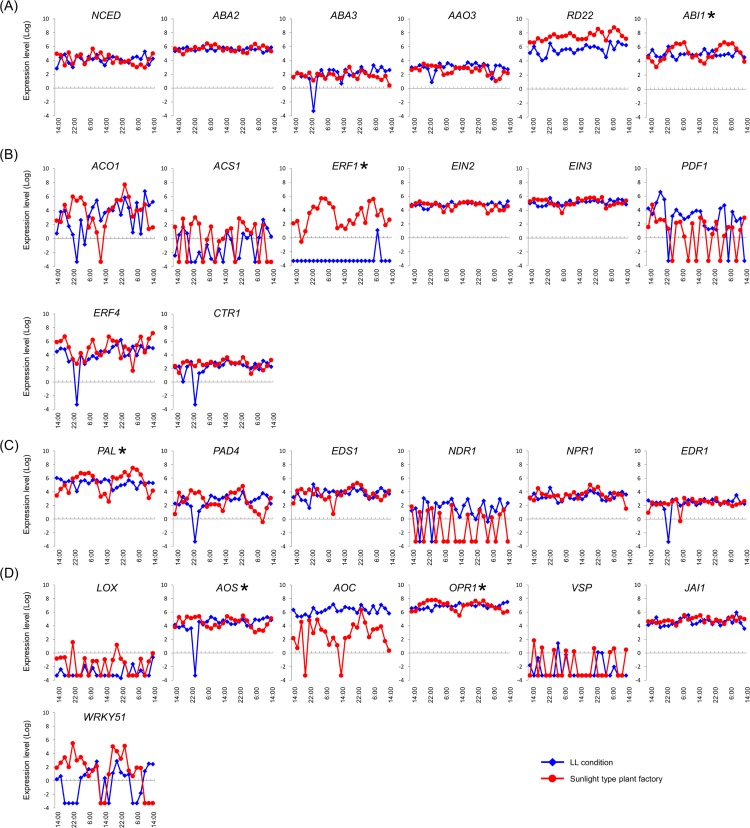
Expression levels of hormone-related tomato genes in the sunlight type factory and under LL conditions. The diamond-shape (blue line) and the circle-shape (red line) indicate expression patterns under LL conditions and in the sunlight type plant factory, respectively. Asterisks (*) indicate temporally expressed hormone-related genes in the sunlight-type plant factory. Temporal expression of hormone-related tomato genes was abolished under LL conditions. (A) Abscisic acid pathway, (B) ethylene pathway, (C) salicylic acid pathway, and (D) jasmonic acid pathway.

To determine the influence of diurnal factors on the temporal expression patterns of hormone-related genes, analyses were performed in tomato plants grown under conditions of continuous light (LL condition). In these experiments, *Sl_AAO3*, *Sl_ERF1*, *Sl_PAL*, *Sl_AOS*, and *Sl_OPR1* lost the temporal expression patterns observed in the sunlight-type plant factory (Fig 9). In particular, the clear temporal expression patterns of *Sl_ABI1* were completely abolished under LL conditions. Moreover, despite loss of temporal patterns, expression levels were maintained over the course of the experiment. Although expression of *Sl_ERF1* in the sunlight-type plant factory was confirmed at all-time points, *Sl_ERF1* expression was not confirmed under LL conditions.

## Discussion

In this study, we analyzed hormone pathway-related genes in tomato plants grown in a sunlight-type plant factory and showed temporal expression of at least one gene for each of four important hormone pathways. Many of these genes had temporal expression patterns that were similar to those in *A*. *thaliana*, and *Sl_ABI1* had the opposite expression pattern to that of *At_ABI1* in both plant species. Whereas, temporal expression patterns of these hormone pathway-related genes were abolished under LL conditions, these data indicate the presence of hormone pathways that are regulated by circadian rhythms independently of light and stress conditions.

The hormone-related genes *Sl_ABI1*, *Sl_ERF1*, *Sl_PAL*, and *Sl_OPR1* had peak expression at 6:00, whereas *Sl_AOS* expression peaked at 0:00. As shown in Fig 1, solar radiation increased from 6:00 on both days and was diminished to about one-third by cloud and rain on the second day. Sustained temporal expression of hormone pathway-related genes suggested that their diurnal regulation may reflect the actions of biological clock genes that maintain circadian rhythms under conditions of continuous dark or light. In the present LL conditions, temporal expression patterns of *Sl_ ABI1*, *Sl_ERF1*, *Sl_PAL*, *Sl_OPR1*, and *Sl_AOS* were abolished, indicating strict regulation by light conditions. Accordingly, growth of tomato plants is inhibited under LL conditions [28]. Sugar metabolism of *A*. *thaliana* is regulated by clock genes independently of photosynthetic activity [5]. Moreover, glucose reportedly promotes degradation of ethylene insensitive 3 and inhibits ET signals [29, 30], and LOXs were regulated by circadian rhythms in a previous study [31]. Taken with these studies, the present data suggest that hormone-related genes of tomato are regulated primarily by light. In addition, it is reported that ABI1 interacts with TOC1 and mutation of TOC1 influenced ABA biosynthesis [13, 27]. Thus, ABI1 is affected by circadian rhythm which produces endogenous rhythm. Finally, PAL has been shown to be regulated by the clock gene [20], suggesting that hormone-related pathways are influenced by circadian rhythms.

In the present study, tomato plants received sun light from 6:00 to 18:00, and whereas *Sl_ABI1* expression decreased, that of *At_ABI1* increased from 6:00. ABI1 and PP2CA are reported direct and indirect suppressors of *OST1* expression [32] and regulate gating of stomata within 1 hour of being expressed [33]. According to these reports, it is expected that tomato stomata open in the evening and close in the morning, reflecting decreased humidity during daylight hours. Under the present sunlight-type plant factory conditions, day-time relative humidity was about 40%, and the relative humidity minimum was 32%, which is drier than that for data from the *A*. *thaliana* diurnal database [34]. Because the tomato plants in this study were grown under dry conditions in the day time, these data suggest that tomato plants do not open stomata to inhibit transpiration and induce ABA synchronously. In addition, we showed that *Sl_TOC1* which affects the ABA biosynthesis had identical expression patterns and *Sl_ABI1* had differential expression patterns compare with *A*. *thaliana*. Moreover, *Sl_OST1* showed the opposite expression pattern to *Sl_ABI1*. It is suggested that the extracellular environment exerts a greater influence on gating of stomata by expression level of ABI1 and OST1. Although differences between tomato and *A*. *thaliana* likely lead to species-specific phenomenon, the expression patterns of ABI1 related genes were predictive of stomata gating in both species. These observations could be exploited in agricultural production to optimize CO_2_ fertilization.

Sustained production quality and quantity are critical for plant factories, warranting further studies of the effects of environmental fluctuations on hormones that are related to growth. In this study, all tomato hormone pathways were regulated by light, and the ET pathway gene *ERF1* was barely detectable under LL conditions, suggesting that ET is more responsive than SA and ABA and may transmit initiating signals to the whole body of the plant and to neighboring plants. These data also indicate that ERF1 could be used as a marker gene for growth stimulation and warrant further investigations.

Because sunlight-type plant factories are sensitive to environmental stresses, early diagnoses of plant abnormalities are critical to food production. The present data show temporal expression of the hormone-related genes *ABI1*, *ERF1*, *PAL*, *AOS*, and *OPR1*, which are central to the production of the plant hormones SA, ABA, ET, and JA. Hence, the expression of these genes could be used to assess the effects of environmental stresses on plants and predict productivity at an early stage without sacrifice of edible tomato tissues. In addition, the present relationships between expression patterns of ABI1 and gating stomata suggest that measurements of humidity could be used to inform effective CO_2_ fertilization and limit stress to plants. Because plant factory can control the humidity easily, we can cultivate an effective plant. In addition, the plant factory can set time for effective fertilization and reduce operation costs. Hence, this study indicates the utility of gene expression analyses for improvements of plant quality at sunlight-type plant factories. Moreover, the present example of plant species was not completely managed, and predictions of plant states were achieved using simple sampling methods and established analytical techniques to facilitate the application of the present analytical techniques to common agricultural monitoring procedures.

## Supporting Information

S1 Table Hormone-related genes from *Arabidopsis thaliana*. Accession numbers were obtained from the NCBI database. ABA, abscisic acid; ET, ethylene; JA, jasmonic acid; SA, salicylic acid.
(DOCX)
