# Correction: Transcriptome Analysis of Plant Hormone-Related Tomato (*Solanum lycopersicum*) Genes in a Sunlight-Type Plant Factory

**DOI:** 10.1371/journal.pone.0150788

**Published:** 2016-03-10

**Authors:** Yusuke Tanigaki, Takanobu Higashi, Kotaro Takayama, Atsushi J. Nagano, Mie N. Honjo, Hirokazu Fukuda

There are errors in the first paragraph of the Materials and Methods section under the heading “RNA-Seq library preparation and sequence analyses.” The correct paragraph is: To prepare RNA-Seq library, the extracted total RNA was mixed with ERCC RNA Spike-In control mixes (Life Technologies, Carlsbad, CA, USA) and then polyA RNA was isolated using oligo(dT)25 Dynabeads (Invitrogen) (see reference 23). Subsequent procedure was according to previously described method (24). Briefly, the purified polyA RNA was fragmented by heat treatment, and reverse transcription was performed using M-MuLV Reverse Transcriptase (Enzymatics) and random hexamers. Second strands were synthesized using DNA polymerase I and RNaseH, and dsDNA ends were repaired using an End-Repair Mix LC (Enzymatics) and dA-tails were added using Klenow 3′→5′ exo- (Enzymatics). A-tailed DNA was added to the Y-shape adapters 5′-A*A*TGATACGGCGACCACCGAGATCTACACTCTTTCCCTACACGACGCTCTTCCGAT*C*T-3′ and 5′-/5P/-G*A*TCGGAAGAGCACACGTC TGAACTCCAGTC*A*C-3′ (asterisk indicates phosphorothioate bonds and 5P indicates phosphorylation) using T4 DNA Ligase (Enzymatics). The ligation product was purified and size selected by using AMpure XP (Beckman Coulter) and then the purified second strand DNA was digested using uracil DNA glycosylase (Enzymatics). DNA fragments with adapters and an index sequence were amplified using a thermal cycler. If small (<200 bp) and large (>600 bp) fragments were remained, they were removed by gel extraction.
