# Supplementary material for: Transcriptome Analysis of Plant Hormone-Related Tomato (Solanum lycopersicum) Genes in a Sunlight-Type Plant Factory
Source: PLoS One. 2015 Dec 1;10(12):e0143412. doi: 10.1371/journal.pone.0143412 (PMC4666670; doi:10.1371/journal.pone.0143412)
Supplement: S1 Table — Accession numbers were obtained from the NCBI database. ABA, abscisic acid; ET, ethylene; JA, jasmonic acid; SA, salicylic acid. (DOCX) [file pone.0143412.s001.docx]

**Table S1.** Hormone-related genes from *Arabidopsis thaliana*

| Pathway | Name | Accession number |
| --- | --- | --- |
| ABA | NCED | AT3G63520 |
|  | ABA2 | AT1G52340 |
|  | ABA3 | AT1G16540 |
|  | AAO3 | AT2G27150 |
|  | RD22 | AT5G25610 |
|  | ABI1 | AT4G26080 |
| ET | ACO1 | AT2G19590 |
|  | ACS1 | AT3G61510 |
|  | ERF1 | AT3G23240 |
|  | EIN2 | AT5G03280 |
|  | EIN3 | AT3G20770 |
|  | PDF1 | AT2G42840 |
|  | CTR1 | AT5G03730 |
|  | ERF4 | AT3G15210 |
| SA | PAL | AT2G37040 |
|  | PAD4 | AT3G52430 |
|  | EDS1 | AT3G48090 |
|  | NDR1 | AT3G20600 |
|  | NPR1 | AT1G64280 |
|  | EDR1 | AT1G08720 |
| JA | LOX | AT1G55020 |
|  | AOS | AT5G42650 |
|  | AOC | AT3G25760 |
|  | OPR1 | AT1G76680 |
|  | VSP | AT5G24780 |
|  | JAI1 | AT1G32640 |
|  | WRKY51 | AT5G64810 |

Accession numbers were obtained from the NCBI database.

ABA, abscisic acid; ET, ethylene; JA, jasmonic acid; SA, salicylic acid.
